# Evaluation of the Clear Fear Smartphone App for Young People Experiencing Anxiety: Uncontrolled Pre– and Post–Follow-Up Study

**DOI:** 10.2196/55603

**Published:** 2025-02-28

**Authors:** Chiara Samele, Norman Urquia, Rachel Edwards, Katie Donnell, Nihara Krause

**Affiliations:** 1 Informed Thinking London United Kingdom; 2 stem4 London United Kingdom

**Keywords:** mental health, anxiety, depression, emotional and behavioral difficulties, mobile phone app, cognitive behavioral therapy, digital tool, young people, mobile phone

## Abstract

**Background:**

Mobile health apps are proving to be an important tool for increasing access to psychological therapies early on, particularly with rising rates of anxiety and depression in young people.

**Objective:**

We aimed to assess the usability, acceptability, safety, and effectiveness of a new app, Clear Fear, developed to help young people manage symptoms of anxiety using the principles of cognitive behavioral therapy.

**Methods:**

The Clear Fear app was developed to provide cognitive behavioral strategies to suit anxiety disorders. An uncontrolled pre– and post–follow-up design over a 9-week period was used to assess the app and its effects. This study comprised 3 phases: baseline (stage 1), post–app familiarization phase (stage 2), and follow-up (stage 3). Eligible participants were aged between 16 and 25 years with mild to moderate anxiety but not currently receiving treatment or in contact with specialist mental health services or using other interventions or apps to help monitor or manage their mental health. A community sample was recruited via advertisements, relevant websites, and social media networks. Eligible participants completed standardized self-report tools and questionnaires at each study stage. These measured probable symptoms of anxiety (7-item Generalized Anxiety Disorder scale) and depression (Mood and Feelings Questionnaire); emotional and behavioral difficulties (Strengths and Difficulties Questionnaire); and feedback on the usability, accessibility, and safety of the app. Mean scores at baseline and follow-up were compared using paired 2-tailed *t* tests or Wilcoxon signed rank tests. Qualitative data derived from open-ended questions were coded and entered into NVivo (version 10) for analysis.

**Results:**

A total of 48 young people entered the study at baseline, with 37 (77%) completing all outcome measures at follow-up. The sample was mostly female (37/48, 77%). The mean age was 20.1 (SD 2.1) years. In total, 48% (23/48) of the participants reached the threshold for probable anxiety disorder, 56% (27/48) had positive scores for probable depression, and 75% (36/48) obtained a total score of “very high” on the Strengths and Difficulties Questionnaire for emotional and behavioral difficulties. The app was well received, offering reassurance, practical and immediate help to manage symptoms, and encouragement to seek help, and was generally found easy to use. A small minority (3/48, 6%) found the app difficult to navigate. The Clear Fear app resulted in statistically significant reductions in probable symptoms of anxiety (*t*_36_=2.6, 95% CI 0.41-3.53; *P*=.01) and depression (*z*=2.3; *P*=.02) and behavioral and emotional difficulties (*t*_47_=4.5, 95% CI 3.67-9.65; *P*<.001), representing mostly medium to large standardized effect sizes.

**Conclusions:**

The Clear Fear app was found to be usable, acceptable, safe, and effective in helping manage symptoms of anxiety and depression and emotional and behavioral difficulties.

## Introduction

### Background

The rates of probable mental health problems in the United Kingdom among young people have steadily increased since 2017. According to National Health Service Digital data (2022), for example, the rates for young people aged 17 to 19 years with mental health problems rose by 7.6% in 2020 and reached 25.7% in 2022 [1]. In 2017 to 2018, depression and anxiety among young women aged 16 to 24 years rose by 26% compared to the previous year [2].

The high volume of young people with experience of symptoms associated with moderate depression and anxiety has prompted a rise in referrals to already overstretched specialist mental health services. For example, in November 2023, a total of 445,000 people were in contact with children and young people’s mental health services compared to 362,000 in November 2021 [3]. New referrals to these services reached a peak of 87,000 per month in 2021, the highest since 2019 [4]. While referrals have increased, there remains a huge shortfall in those who need support and receive it. In 2020, the proportion of children and young people needing and receiving support was 27% [4].

Therefore, it is important to find ways to ensure that young people have timely access to support. One growing avenue is support through mobile health apps.

Approximately 96% of those aged between 16 and 24 years own a smartphone [5]. Mobile health apps now constitute an important tool for increasing access to psychological therapies, particularly for young people. Several published systematic reviews have assessed the usability, feasibility, acceptability, functionality, and effectiveness of specific mobile mental health apps for young people [6,7]. Despite the published literature on how mobile app technology can increase access to psychological therapy, relatively little research exists on its effectiveness and whether it improves symptoms of mental health problems. However, there is some evidence suggesting that certain mobile apps for mental health can help improve the monitoring and management of mental health [8]. A handful of studies confirm that web-based cognitive behavioral therapy (CBT) is as effective as face-to-face treatment for anxiety and depression [9]. Among young people, mobile mental health apps also offer an important opportunity to prevent mental health problems or intervene early to reduce the risk of a long-term mental health condition into adulthood [10].

### Objectives

Given the limited available evidence on the effectiveness of mental health apps for young people, this pilot study aimed to assess whether the Clear Fear app, developed to help young people manage symptoms of anxiety, could reduce these symptoms.

The main aims of this study were to assess the usability, acceptability, safety, and effectiveness of the Clear Fear app, specifically (1) what particular features of the app young people found most helpful or beneficial; (2) the ease of use, acceptability, and frequency with which young people used the app, as well as whether they found it safe and any improvements that could be made; and (3) whether the Clear Fear app was able to reduce or improve symptoms of anxiety primarily (and any adverse behavior) following its use during the study period.

Thus, this study was designed to test the following hypothesis: a self-guided mobile cognitive behavioral app can improve anxiety symptoms and be acceptable to young people over a period of 9 weeks.

## Methods

### Clear Fear App

#### Aims of the App

The Clear Fear app aims to support young people in managing symptoms of anxiety early in their stage of development. It does this by (1) providing cognitive behavioral strategies to suit anxiety disorders; (2) encouraging self-monitoring and self-management and building resilience; and (3) teaching alternative, adaptive ways to deal with anxiety.

Users manage their anxiety and “face their fear” in a gradual way while learning to manage panic, challenge worries, and regulate their emotions.

The app provides a first step for young people looking for immediate techniques to help manage anxious thoughts and behaviors for longer-term change. It was also designed to be easily accessible for those who do not meet the criteria for mental health services or are currently on a waiting list to receive appropriate treatment for anxiety disorder. The app does not replace specialist treatment but can be used in addition to it.

#### Development, Structure, and App Function

The app was developed by NK at stem4, a charity supporting teenage mental health, in collaboration with young people. The development process began with reviewing available mainstream apps that aimed to manage anxiety through mindfulness. Integral to the process was the coproduction of the Clear Fear app with a group of 30 young people (aged between 14 and 17 years) who took part in a series of user engagement sessions to explore desired outputs, user journeys, visual concepts, colors, imagery, security and privacy considerations, user experience, and tone of voice. The app provides a low-intensity CBT treatment to monitor and change thoughts, emotions, and behaviors associated with mild to moderate symptoms of anxiety. It seeks to calm fear responses through a cognitive behavioral framework along with breathing, relaxation, and mindfulness exercises.

**Figure 1:**
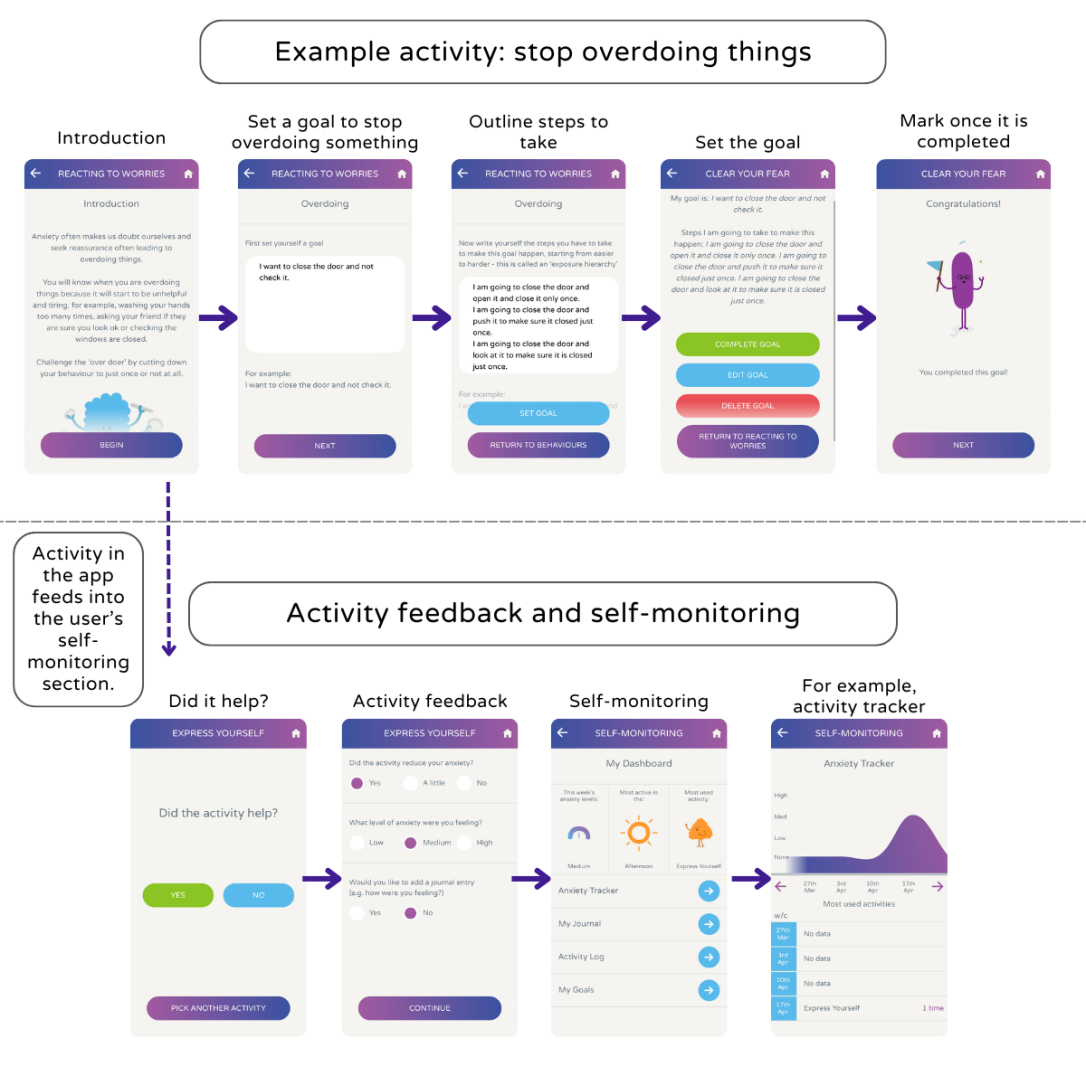
Flowchart and screenshots of the Clear Fear app, the digital intervention used in this uncontrolled pre– and post–follow-up study to evaluate its usability, acceptability, safety, and effectiveness in helping young people manage symptoms of anxiety using the principles of cognitive behavioral therapy over a 9-week period.

The Clear Your Fear section of the app provides activities to help users with the 4 key areas—dealing with emotions, managing worries, reacting to worries, and managing physical responses to anxiety—and is described in Textbox 1 and Figure 1. Each activity provides some brief introductory information to explain why the activity can help, as well as examples where necessary throughout.

The multiple areas for goal setting provided in the app facilitate the following: identifying specific goals, tailoring each goal to suit each individual, identifying barriers, problem-solving, breaking long-term goals into short-term challenges, accomplishing easier challenges first, prompts, cueing, reinforcing success, social support, and tracking personal information to enhance self-directed learning.

**Description of the Clear Your Fear section of the Clear Fear app.**

**Dealing With Your Emotions**

This consists of activities for users to learn to identify, express, and regulate emotions, which can help reduce anxiety.

- Express Yourself activities provide opportunities for note keeping and finding alternative ways of emotional regulation.
- Stay Calm activities help users learn to calm the body and mind by doing either the guided breathing activity or mindfulness exercises.
- Laugh & Smile activities aid in substituting negative emotions for positive ones through the use of humor by viewing jokes or gifs.

**Managing Your Worries**

This focuses on learning how thinking can contribute to anxiety and how to make a change by learning to understand, identify, and change fearful thoughts (Worry Warriors); have a break from anxious thoughts (Worry Box); or put a problem-solving strategy into practice (Worry Ladder).

- Worry Warriors guides the user through a process of cognitive restructuring to identify and combat 3 common types of automatic negative thoughts (those that have been magnified by anxiety or taken to the extreme or that are about something negative that is expected to happen), together with self-monitoring, seeking evidence, and restructuring thoughts. The resulting positive thoughts that are created can then be revisited in the Grit Box, located in another area of the app. Therapeutic gains from this process can include symptom relief and an increased sense of control over anxiety through its objectification.
- Worry Box is an activity in which the user types in their worries to be stored in this section of the app, which can then be emptied to metaphorically clear them.
- Worry Ladder guides the user through dealing with their worries one step at a time (and creating a visual ladder of these steps in the process) by first stating a worry and then typing in possible solutions; choosing one to try; and, finally, noting what happened and what worked. Both the Worry Ladder and Worry Box, together with problem-solving, help with prioritizing and containing anxiety.

**Reacting to Worries**

This involves the user identifying situations in which they want to experience less anxiety and learn to face their worries with the help of activities to lessen overdoing or avoiding things. In each case, the user is guided through setting a goal and breaking it down into an exposure hierarchy of manageable steps and can also mark when a goal has been achieved.

**Managing Physical Responses to Anxiety**

This consists of activities for users to cope with and lessen the physical effects of anxiety. In total, 4 categories of goals are presented (exercise, diet, relaxation, and sleep), within which the user is given brief information as to why the activity can help and suggestions of goals to set, which can be input into the app. The user is also given the option of setting push notifications (at daily, weekly, or monthly intervals) for the goal to help remind them.

#### Activity Feedback and Self-Monitoring

Upon completion of an activity, the user is asked to provide the following feedback: whether the activity helped (Yes or No), whether it reduced their anxiety (Yes, A little, or No), and the level of anxiety they were feeling (Low, Medium, or High). This information feeds into that user’s self-monitoring section on the app, which includes an anxiety profile and anxiety tracker to help the user self-monitor across every task.

#### Grit Box

The Grit Box is based on the concept that grit is a necessary factor in resilience. It holds the user’s own Worry Warrior Positive Thoughts, which are generated from their activity on the app. It also contains a range of motivational statements, positive quotes, and information on resilient and inspirational people as suggested during the coproduction process with young people.

#### Other Areas of the App

Psychoeducation is provided in the information section and in optional notifications sent to the users to support their momentum in reaching personal goals and in gaining valuable psychological insights (eg, setting realistic goals, overcoming fears, and increasing motivation). On achieving a goal, users receive a plan on how to maintain the new skills and avoid falling back into old habits.

In addition, information about anxiety is provided via messages sent to the user to help normalize their experience, as well as giving information on the value and functioning of anxiety. This includes information on specific types of anxiety (namely, generalized anxiety, social anxiety, performance anxiety, obsessive-compulsive disorder and health anxiety, separation anxiety, exam anxiety, phobias, and fear of missing out).

Finally, users can receive physiological support and help in dealing with panic and panic attacks based on mindfulness exercises in both visual and auditory formats to improve low self-esteem, emotional reactivity, and functioning [11], as well as signposts to further support.

#### The Clear Fear App’s Contribution to Digital Mental Health

The Clear Fear app is a clinician-developed, evidence-based, and comprehensive CBT app that differs from those using mindfulness and meditation as their primary interventions, artificial intelligence chatbots, and other available apps for anxiety management developed *without* clinician input or comprehensive evidence-based approaches [12,13].

The Clear Fear app is designed primarily for managing anxiety symptoms in young people. It provides a comprehensive evidence-based approach that is both structured and personalizable, incorporating CBT exercises, psychoeducation, mindfulness, and self-monitoring. The content, language, visuals, tone of voice, and user journeys are age appropriate. For younger users aged between 11 and 15 years, the Clear Fear app includes age-appropriate design and language, ease of navigation, age-relevant gamification, less abstract cognitive concepts, tasks that cater to shorter attention spans, and increased socialization.

It also prioritizes user privacy and data safety as it does not collect identifiable information or require user accounts, can be passcode protected, is regularly reviewed and updated, and is compliant with regulatory standards such as the National Health Service clinical safety standard DCB0129.

Unlike many mental health apps, Clear Fear is free to download in the United Kingdom, with no subscriptions, in-app purchases, or advertisements, and can be used without an internet connection, making it highly accessible.

### Study Design

This study used an uncontrolled pre– and post–follow-up design over a 9-week period to assess the app and its effects. The study comprised three main phases: (1) baseline and app introduction (stage 1), (2) post–app familiarization phase (stage 2), and (3) follow-up (stage 3).

### Participants and Eligibility Criteria

Participants were a community sample recruited via stem4’s website and their existing social media networks.

Potential participants were eligible to take part in the study if they (1) were aged between 16 and 25 years and (2) had mild to moderate anxiety.

Participants were excluded if they were (1) in contact with mental health services or taking medication for a mental health problem; (2) using other interventions or apps to monitor their mental health; or (3) at risk of suicide, diagnosed with a severe and enduring mental health problem such as psychosis or major depression, or experiencing substance use problems.

Potential participants were screened using a 10-item questionnaire developed for this study on suicide ideation, any severe mental health problems, and substance use issues (drugs and alcohol).

### Recruitment

The main method for recruiting participants was through advertisements on the stem4 website and via stem4 social media channels inviting young people who experienced anxiety and had not previously used the Clear Fear app to take part in the study. stem4 also provided details of the study to schools within their local area. Eligible participants were offered a web-based shopping voucher as an incentive to take part in the study.

A web-based screening questionnaire (developed for this study) was sent to all young people interested in taking part in the study to assess their study eligibility and level of risk. This included a 10- to 15-item screening tool to identify any current mental health issues or history of and contact with mental health services. Those identified with an increased risk at initial screening were excluded from the study to prioritize their safety. These individuals were provided with an information pack containing emergency contact numbers and guidance on further support, and they were encouraged to notify the research team if they accessed additional support.

Eligible participants were provided with an information sheet detailing the study and asked to complete an informed consent form before taking part. Participants could leave the study at any time during the study without giving a reason if they wished. Participants were also made aware that the Clear Fear app does not replace traditional treatment and were appropriately signposted to sources of help where necessary. Participants also received an introductory leaflet with information on symptoms and suggested strategies for mitigating risk and were directed to sections on the app that provided immediate signposts and access to services.

Furthermore, at the start of the study, all participants completed the Safety Net section of the app, which is a vital safety feature that is always available to the user and can be shared with responsible adults. The users populated what they could think and do when they needed help and the numbers of people they could contact to go along with the Safety Net section’s prepopulated emergency numbers.

The app also prompted users to seek help from a trusted adult or general practitioner if they noticed that their anxiety levels, from the tracker in the Self-Monitoring section, were consistently high.

### Sample Size

The aim was to recruit approximately 45 young people to address the main study aims. No formal sample size calculation was made given the preliminary nature of the study and piloting of the Clear Fear app. We allowed for attrition rates of up to 15% at the postfamiliarization and follow-up time points.

### Study Procedure

**Figure 2:**
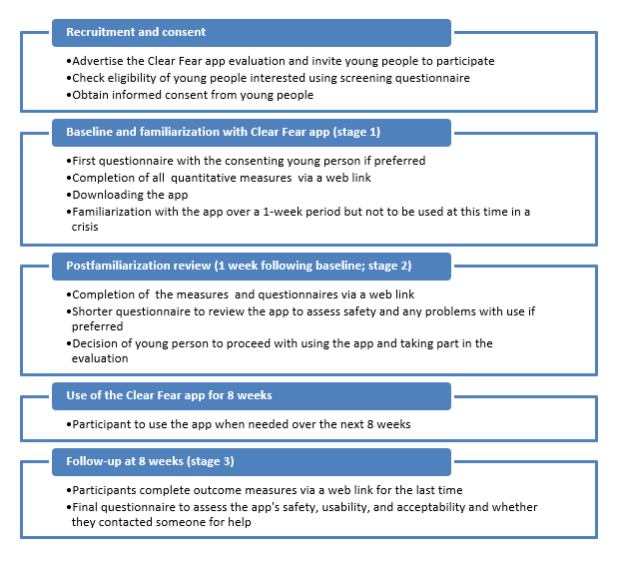
Diagram of the procedure of this uncontrolled pre– and post–follow-up study to evaluate the Clear Fear app’s usability, acceptability, safety, and effectiveness in helping young people manage symptoms of anxiety using the principles of cognitive behavioral therapy over a 9-week period.

Figure 2 shows the study procedure, including the 3 main study stages and the recruitment process. We also consulted with 6 people (aged between 14 and 17 years) to provide feedback on the study proposal and procedure regarding their preferences concerning web-based data collection.

#### Baseline (Stage 1)

Upon entry to the study, participants were provided with web-based instructions on how to download and use the Clear Fear app and the study procedure. Participants were then allocated a study ID number to maintain confidentiality and anonymity and emailed a link to the web-based baseline questionnaires. The link was created by the researchers using SurveyMonkey (SurveyMonkey Inc).

At this stage, participants were asked only to familiarize themselves with the Clear Fear app over the coming week and not to use it during a time of crisis. This was to ensure the person’s safety until they became fully versed in using the Clear Fear app and to obtain baseline measures of the frequency and severity of anxiety they experienced and their existing coping mechanisms.

#### Post–App Familiarization Phase (Stage 2)

One week after baseline, web-based questionnaires were sent again to participants, which included a user questionnaire to gauge their assessment of the Clear Fear app and any problems they may have had. If the participant wished to continue taking part in the study, they were asked to complete the outcome measures again. Participants were informed that they could now use the Clear Fear app as often as they wanted over the following 8 weeks.

#### Follow-Up (Stage 3)

After an 8-week period of using the Clear Fear app, participants were sent the web-based questionnaires for a final time to complete, again including the user questionnaire for their specific feedback on using the app. In total, 2 to 3 attempts were made to follow up on participants if no response was received. If no response was received, it was assumed that the participant had withdrawn from the study.

#### Ongoing Monitoring and Risk Assessment

Throughout the study, participants self-reported their anxiety levels at regular intervals. If they reported high levels of anxiety or emerging new symptoms, they were encouraged to consult a general practitioner or, depending on the severity, seek more urgent help. After the study, participants were given resources on maintaining well-being.

stem4, the owners of the app, had a clinical safety protocol and a hazard mitigation list that was managed and reviewed regularly with clear timelines of response. Their clinical safety officer was available to respond to inquiries daily.

#### Data Collection

A standardized questionnaire was developed to gather the eligible participants’ demographic characteristics. To assess the Clear Fear app and its effects, participants were asked to complete 3 outcome measures to assess their emotional state or mood and behavior and the Clear Fear user questionnaire. Participants completed these 4 measures at stages 2 (post–app familiarization phase) and 3 (follow-up).

#### Outcome Assessments

In total, 3 self-report outcome measures were administered to participants to assess their symptoms of anxiety (main outcome) and depression and their behavior.

For anxiety, the 7-item Generalized Anxiety Disorder scale (GAD-7) was used, covering various areas, including social phobia, anxiety, and obsessive-compulsive disorder [14]. Total scores of 5, 10, and 15 indicate probable mild, moderate, and severe anxiety, respectively.

For depression, the 13-item self-report Mood and Feelings Questionnaire (MFQ) was used, with item ratings of *true* (score of 2), *sometimes true* (score of 1), or *not true* (score of 0) [15]. A total score of ≥12 may indicate depression.

For emotional and behavioral difficulties, the Strengths and Difficulties Questionnaire (SDQ) was used. This is a 25-item questionnaire to measure behavioral and emotional difficulties and assess psychological adjustment and mental health problems in children and young people [16].

The child and adolescent versions of the MFQ and the SDQ were used for participants aged between 16 and 17 years, and the adult version was used for those aged ≥18 years.

#### Clear Fear App Questionnaire

The Clear Fear app questionnaire, developed for the purposes of this study, was used to assess the Clear Fear app’s safety, acceptability, satisfaction, and usability and included 21 items (of which 7 were closed ended, 9 were open ended for free-text responses, and 5 had Likert scales from 1-5).

For acceptability and user satisfaction, 10 items, including 8 open-ended questions, asked about what parts of the Clear Fear app were used, whether the content was useful, what features were liked or disliked and why, whether the app met the participants’ expectations, and whether the app was unloaded.

For safety, 4 items asked whether the app helped reassure, reduce, or increase feelings of anxiety and whether the participants had to contact someone.

For usability, 7 items were related to how easy the Clear Fear app was to use, whether any technical issues were encountered, frequency of use, and at what time of the day it was used.

#### Data Analysis

Statistical analyses for all quantitative data were conducted using SPSS Statistics for Windows (version 29; IBM Corp). The demographic profile of participants and other quantitative data were described using frequencies, percentages, and means. The Shapiro-Wilk test of normality and visual assessment of *Q*-*Q* plots were used to check whether continuous variables for the outcome scores were normally distributed.

Mean scores derived from the outcome measures (GAD-7, MFQ, and SDQ) were compared between baseline and follow-up. Tests of significance were conducted using 2-tailed *t* tests or Wilcoxon signed rank tests (for continuous variables not distributed normally) to compare baseline and follow-up scores for all outcome and clinical measures. Standardized effect sizes were calculated using the Cohen *d* based on the mean change in outcome scores between baseline (stage 1) and follow-up (stage 3).

Qualitative data derived from open-ended questions were coded and entered into NVivo (version 10; QSR International) and analyzed using a thematic analysis of the text to identify themes and patterns in the data and how they addressed the main aims of the study [17]. Additional analyses (means and SDs) were conducted to examine any differences in total outcome scores for the GAD-7 and MFQ among age, gender, and ethnic groups. Differences among these groups were also explored through qualitative feedback on the Clear Fear app’s acceptability using thematic analysis and categorization according to age, gender, and ethnic group.

### Ethical Considerations

Ethics approval for this study was obtained from the London – City and East Research Ethics Committee (United Kingdom; 20/PR/0268), and all practices and procedures throughout the research process were in line with the stipulations of the Declaration of Helsinki.

This included that eligible participants be suitably informed of the study details, risks, and benefits via an information sheet before being asked to consent to the study. Only those who thus provided their informed consent went on to enter the study.

Participants’ data were anonymized, collected and stored securely, and managed solely by the researchers. Participants were allocated a study ID number by a project administrator separate from the researchers.

Finally, participants were given a £20 (US $24.44) Amazon voucher as an honorarium upon completion of the final stage.

## Results

### Overview

Recruitment for the study took place between February 2021 and June 2022. A total of 48 young people completed the baseline measures. At the postfamiliarization phase (stage 2), 92% (44/48) of the participants completed all measures and the Clear Fear app user questionnaire. At the 8-week follow-up (stage 3), 77% (37/48) of the participants submitted all required responses, constituting a dropout rate of 23% (11/48).

### Demographic Characteristics

The demographic characteristics of the sample are listed in Table 1. The mean age of the sample was 20.1 (SD 2.1) years, with 62% (30/48) aged between 18 and 25 years. The sample was predominantly female (37/48, 77%). Most were students, with a third (16/48, 33%) attending secondary school and 29% (14/48) at university. In terms of ethnicity, 67% (32/48) were White British, and 10% (5/48) were Black British.

**Table 1:** Characteristics of the participants at baseline (stage 1) of this uncontrolled pre– and post–follow-up study to evaluate the Clear Fear app’s usability, acceptability, safety, and effectiveness in helping young people manage symptoms of anxiety using the principles of cognitive behavioral therapy over a 9-week period (N=48).

Characteristics			Values
**Age (y), mean (SD; range)**			20.1 (2.1; 17-24)
	16-17, n (%)	18 (38)	
	18-25, n (%)	30 (62)	
**Sex, n (%)**			
	Female	37 (77)	
	Male	11 (23)	
**Marital status, n (%)**			
	Single	41 (85)	
	Married or cohabiting	4 (8)	
	Other long-term relationship	3 (6)	
**Ethnicity, n (%)**			
	Asian or Asian British	2 (4)	
	Black British	5 (10)	
	Mixed heritage	3 (6)	
	White British	32 (67)	
	White other	3 (6)	
	Other	3 (6)	
**Student status, n (%)**			
	Attending secondary school	16 (33)	
	Attending university	14 (29)	
	Attending sixth form or college	10 (21)	
	Not currently a student	8 (17)	
**Employment status, n (%)**			
	Student	33 (69)	
	Employed part time	6 (12)	
	Employed full time	5 (10)	
	Unemployed	2 (4)	
	Other	2 (4)	

### Baseline Scores

Table 2 lists the numbers and percentages of participants with probable symptoms of anxiety and depression and emotional and behavioral difficulties at baseline that would require further assessment. In total, 48% (23/48) of the participants reached the threshold for probable anxiety disorder as measured using the GAD-7, with 29% (14/48) finding it *very difficult* or *extremely difficult* to go about their daily routine or get along with others.

On the basis of the short MFQ measure, 56% (27/48) of the participants obtained a positive score for probable depression. A total of 75% (36/48) obtained *very high* on the SDQ total score, with 52% (25/48) of participants reporting their problems to be considerably distressing (*quite a lot* or *a great deal*). The ability to learn (23/48, 48%) and get along with others close to them (21/48, 44%) was also impacted for many.

**Table 2:** Numbers and percentages of participants meeting the threshold for probable symptoms of anxiety and depression and emotional and behavioral difficulties at baseline (stage 1) and follow-up (stage 3) following use of the Clear Fear app over a 9-week period to evaluate the app’s usability, acceptability, safety, and effectiveness in helping young people manage symptoms of anxiety using the principles of cognitive behavioral therapy.

			Baseline (stage 1; n=48), n (%)		Follow-up (stage 3; n=37), n (%)
Generalized anxiety disorder (GAD-7^a^)^b^			23 (48)		8 (22)
Mood and feelings (short MFQ^c^)^d^			27 (56)		8 (22)
**Emotional and behavioral difficulties (SDQ^e^)^f^**			36 (75)		23 (62)
	Definite or severe problem	19 (40)		10 (27)	
	Problem lasting >12 months	19 (40)		14 (38)	
	Problem causing distress^g^	25 (52)		12 (32)	
	Difficulties getting along with close family or friends	21 (44)		14 (38)	
	Difficulties making and keeping friends	14 (29)		12 (32)	
	Difficulties with learning, studying, or work	23 (48)		17 (46)	
	Difficulties with hobbies and leisure	14 (29)		13 (35)	
	Difficulties impacting on others	19 (40)		6 (16)	

^a^GAD-7: 7-item Generalized Anxiety Disorder scale.

^b^Total score of ≥10.

^c^MFQ: Mood and Feelings Questionnaire.

^d^Total score of ≥12.

^e^SQD: Strengths and Difficulties Questionnaire.

^f^Total score of 20 to 40 (*very high*).

^g^Those reporting quite a lot to a great deal.

### Postfamiliarization Phase (Stage 2): Clear Fear App Use

A total of 92% (44/48) of the participants completed the self-report measures and questionnaires at stage 2. In total, 77% (34/44) of the participants used iOS (iPhone) as their operating system, with the remainder (10/44, 23%) using an Android phone. Only 5% (2/44) of the participants encountered technical issues when using the Clear Fear app (eg, poor display or phone freezing).

Participants used the Clear Fear app quite frequently; for many, it was several times a week (18/44, 41%) or every day (11/44, 25%). The evening was the preferred time for over half (24/44, 55%) of the participants.

Commonly used parts of the Clear Fear app included Anxiety Types (25/44, 57%), Clear Your Fear (23/44, 52%), and Self-Monitoring (20/44, 45%; Table 3). Parts of the Clear Fear app that were used less included Express Yourself (6/44, 14%), Laugh And Smile (6/44, 14%), information (6/44, 14%), Stop Overdoing Things (4/44, 9%), and Set an Exercise Goal (4/44, 9%).

**Table 3:** Participants’ use of the Clear Fear app at the postfamiliarization phase (stage 2; 1 week following baseline) of this uncontrolled pre– and post–follow-up study to evaluate the app’s usability, acceptability, safety, and effectiveness in helping young people manage symptoms of anxiety using the principles of cognitive behavioral therapy over a 9-week period (N=44).

			Participants, n (%)
**Frequency of use**			
	More than once a day	2 (5)	
	Every day	11 (25)	
	Several times a week	18 (41)	
	Weekly	10 (23)	
	Less than once a week	3 (7)	
**Parts of the app used most**			
	Anxiety Types	25 (57)	
	Clear Your Fear	23 (52)	
	Self-Monitoring	20 (45)	
	Stay Calm	14 (32)	
	Dealing With Your Emotions	13 (30)	
	Managing Your Worries	12 (27)	
	Safety Net	12 (27)	
	Combat Negative Thoughts	10 (23)	
	Immediate Help	8 (18)	
	Managing Physical Responses to Anxiety	8 (18)	

### Follow-Up (Stage 3)

#### Usability and Acceptability

##### Overview

At follow-up (stage 3), 77% (37/48) of the participants completed the Clear Fear app user questionnaire. A total of 70% (26/37) of the participants reported using the Clear Fear app to address and manage feelings of anxiety, panic attacks, fear, stress, and worry. Participants were asked what they did before using the Clear Fear app to manage these symptoms. Most (23/37, 62%) used positive distractions, such as talking to family members, exercising, or other activities. The remaining participants either did nothing or used negative distractions such as “be in a daze.”

The use pattern of the Clear Fear app was similar to that in stage 2 as the most used parts continued to be Anxiety Types (20/37, 54%), Clear Your Fear (24/37, 65%), and Self-Monitoring (16/37, 43%) and the least used parts were Express Yourself (4/37, 11%) and Stop Overdoing Things (4/37, 11%). Participants again mostly used the Clear Fear app either several times a week (17/37, 46%) or weekly (14/37, 38%) and in the evening (15/37, 41%). Fewer participants reported using the Clear Fear app every day at stage 3 compared to stage 2 (4/37, 11% vs 11/44, 25%, respectively). Most participants (31/37, 84%) found the Clear Fear app easy or very easy to use.

In total, 4 key themes were derived from the qualitative data (open-ended text responses). These included the Clear Fear app’s interactive approach, active exercises, navigation, and app look and content.

##### Interactive Approach

When asked what features of the Clear Fear app were liked the most and why, a common theme appeared to include its interactive and direct approach while simultaneously providing information and guidance on how to manage feelings of anxiety and fear:

I liked the way the app asked what you were afraid of and then what you could do to battle that. I liked the way the questions made you face your fears and really think about it and not hide them.

P21; female; student; aged 20 years

This interactive approach gave one participant a sense of being more in control:

I like things where I actively did something with my anxiety, such as putting worries away, breathing exercises, clear fear, etc. The active involvement made me feel more in control.

P59; female; volunteer; aged 24 years

##### Active Exercises

The range of different activities, including breathing and writing exercises, to guide participants through their negative thoughts and feelings was well received. The immediate help feature and the available activities were considered helpful:

I liked using the immediate help feature and doing the breathing activities and panic attack feature because it helped to calm me down while having a panic attack. I also liked the safety net feature to remind myself what help I could get.

P65; female; student; aged 18 years

Encouraging participants to seek help when they needed it was another important feature of the Clear Fear app, together with offering practical and immediate strategies to manage their anxiety.

##### Navigation

The app is generally great and easy to use, and very helpful.

P61; male; working part time; aged 21 years

While most participants found the Clear Fear app easy to use and follow, a small minority (5/37, 14%) found the Clear Fear app difficult to use. A total of 60% (3/5) of these participants found the app difficult to navigate and asked for clearer instructions for finding activities:

The app was a little confusing to navigate on the home page, I found it confusing to go to the activities because there was no instruction as to how to do that.

P32; female; student; aged 17 years

Some features take a long time to find and I forgot which buttons I needed to press to get to them.

P35; female; student; aged 17 years

Another 40% (2/5) mentioned that they wanted to be able to make entries to the journal part of the app without having to carry out the activities first:

I couldn’t find a way to always access the journal. My entries were after activities where it gives you the option to write. I’d love there to be a more accessible journal.

P59; female; other; aged 22 years

In total, 2 features of Clear Fear, Grit Box and Laugh and Smile, were considered less helpful by a small minority (4/37, 11%).

##### App Look and Content

While the Clear Fear app had content that participants found useful, some preferred not to have to read so much. One participant described expecting the Clear Fear app to have a better layout and look, with a less clinical appearance:

I expected the physical layout and colour schemes and fonts to look more calming and relaxing. The white backgrounds and lack of obvious clickable buttons made it look very clinical. To some extent the confusing layout made me feel more anxious when I used it.

P29; female; student; aged 18 years

Another participant thought that the Clear Fear app would have a means through which to communicate with someone or something:

I thought that there would be some sort of communication with something or someone like an area where you can talk to those cute creature things and have them say nice messages.

P65; female; working full time; aged 21 years

There was also a suggestion to have the Clear Fear app speak, perhaps to offer reassurance or include games:

I wanted more options of help than just writing everything down and having to read the help. E.g. videos to watch on the topic, music to calm thoughts and someone speaking the words.

P30; female; student; aged 18 years

#### Safety

Table 4 lists the numbers and percentages of participants concerning their responses to questions about the Clear Fear app’s safety. Many participants found that the Clear Fear app reassured them either *a lot* (11/37, 30%) or *somewhat* (16/37, 43%). Similarly, the Clear Fear app was said to help reduce feelings of anxiety by *a lot* (13/37, 35%) or *somewhat* (14/37, 38%).

**Table 4:** Participants’ responses regarding the safety of the Clear Fear app following 8 weeks of use (at stage 3) in this uncontrolled pre– and post–follow-up study to evaluate the app’s usability, acceptability, safety, and effectiveness in helping young people manage symptoms of anxiety using the principles of cognitive behavioral therapy (N=37).

Questions and responses			Participants, n (%)
**Did the Clear Fear app help reassure you when feeling anxious?**			
	Yes, a lot	11 (30)	
	Yes, somewhat	16 (43)	
	Yes, a little	5 (14)	
	No, not at all	2 (5)	
**Did the Clear Fear app reduce your feelings of anxiety?**			
	Yes, a lot	13 (35)	
	Yes, somewhat	14 (38)	
	Yes, a little	7 (19)	
	No, not at all	2 (5)	
**Did the Clear Fear app help you contact someone to speak to about your anxiety?**			
	Yes	21 (57)	
	No	16 (43)	

Just over half (21/37, 57%) of the participants who responded to this question reported that the Clear Fear app helped them speak to someone about their anxiety. However, 43% (16/37) said that the Clear Fear app did not help them contact someone.

#### Qualitative User Feedback and Differences by Age, Gender, and Ethnicity

Some differences were found among age, gender, and ethnic groups from the qualitative data gathered from 3 user feedback questions concerning the acceptability of the Clear Fear app—which parts were most liked and disliked and what users were expecting from the app. Younger users (aged 16-18 years) preferred guided, calming features such as Clear Your Fear, whereas older users (aged 19-25 years) appreciated action-oriented, self-reflective tools such as the worry box and anxiety management features. The small sample of men focused on more immediate relief tools, such as breathing exercises and the Laugh and Smile feature, whereas female participants opted more for features that allow for emotional processing (eg, writing and emotional guidance). Regarding ethnicity, Black British users highlighted a focus on privacy and control, and Asian British participants valued ease of use and humor.

There were few differences between demographic groups for the small number of participants who noted parts of the app that they disliked (5/37, 14%). Younger users (aged 16-18 years) appeared less satisfied with navigating the app and features such as Safety Net or Laugh and Smile. Male respondents were less dissatisfied with the Clear Fear app, although they provided very brief responses. Black British users found the app’s interface and navigation difficult and preferred customizable features, whereas Asian British participants provided limited negative feedback.

Managing emotions, anxiety, and coping strategies were the main expectations from the app across each of the age, gender, and ethnic groups.

#### Effectiveness of the Clear Fear App: Outcomes at Follow-Up (Stage 3)

Tables 5 and 6 list mean scores (and subscores for the SDQ), mean differences, standardized effect sizes, 95% CIs, and *P* values for each outcome measure at baseline and follow-up. At follow-up, 8 weeks after stage 2, statistically significant reductions were found in all mean total scores for symptoms of anxiety (GAD-7; *t*_36_=2.6, 95% CI 0.41-3.53; *P*=.01) and depression (short MFQ; *z*=2.3; *P*=.02) and emotional and behavioral difficulties (SDQ; *t*_47_=4.5, 95% CI 3.67-9.65; *P*<.001). The mean change in mean symptom scores revealed mostly medium to large standardized effect sizes.

Subscores for the SDQ also revealed significant decreases in emotional problems (*t*_47_=4.5, 95% CI 1.14-2.99; *P*<.001), conduct problems (*t*_47_=4.2, 95% CI 0.64-1.82; *P*<.001), hyperactivity (*t*_47_=2.3, 95% CI 0.11-1.59; *P*=.02), and peer problems (*t*_47_=1.6, 95% CI 0.39-1.61; *P*=.002).

**Table 5:** Baseline and follow-up (9 weeks later) mean comparisons, mean differences, and standardized effect sizes for participants’ anxiety, depression, and behavioral and emotional symptom scores following their use of the Clear Fear app in this uncontrolled pre– and post–follow-up study to evaluate the app’s usability, acceptability, safety, and effectiveness in helping young people manage symptoms of anxiety using the principles of cognitive behavioral therapy.

Outcome measure		Baseline score (stage 1; n=48), mean (SD)	Follow-up score (stage 3; n=37), mean (SD)	Mean difference (SD)	Standardized effect size, Cohen *d*_*z*_
Probable anxiety disorder (GAD-7^a^ total)		9.4 (4.5)	7.5 (3.7)	–1.9 (4.1)	0.46
Probable depression (short MFQ^b^ total)		11.3 (5.4)	8.7 (5.1)	–2.6 (5.2)	0.50
**Behavioral and emotional difficulties**		23.3 (4.8)	16.6 (9.9)	–6.7 (8.3)	0.80
	Emotional problems	5.9 (2.1)	3.9 (2.9)	–2.0 (2.5)	0.80
	Conduct problems	2.7 (1.8)	1.5 (1.2)	–1.2 (1.5)	0.79
	Hyperactivity	4.1 (1.9)	3.3 (2.7)	–0.8 (2.3)	0.34
	Peer problems	3.1 (1.8)	2.1 (1.9)	–1.0 (1.8)	0.54

^a^GAD-7: 7-item Generalized Anxiety Disorder scale.

^b^MFQ: Mood and Feelings Questionnaire.

**Table 6:** Baseline and follow-up (9 weeks later) test of significance of the results for participants’ anxiety, depression, and behavioral and emotional symptom scores following their use of the Clear Fear app in this uncontrolled pre– and post–follow-up study to evaluate the app’s usability, acceptability, safety, and effectiveness in helping young people manage symptoms of anxiety using the principles of cognitive behavioral therapy.

Outcome measure			Test of significance		*P* value
Probable anxiety disorder (GAD-7^a^ total)			*t*_36_=2.6 (95% CI 0.41-3.53)		.01
Probable depression (short MFQ^b^ total)			*z*=2.3^c^		.02
**Behavioral and emotional difficulties (SDQ^d^ total)**			*t*_47_=4.5 (95% CI 3.67-9.65)		<.001
	Emotional problems	*t*_47_=4.5 (95% CI 1.14-2.99)		<.001	
	Conduct problems	*t*_47_=4.2 (95% CI 0.64-1.82)		<.001	
	Hyperactivity	*t*_47_=2.3 (95% CI 0.11-1.59)		.02	
	Peer problems	*t*_47_=1.6 (95% CI 0.39-1.61)		.002	

^a^GAD-7: 7-item Generalized Anxiety Disorder scale.

^b^MFQ: Mood and Feelings Questionnaire.

^c^*Z* score from Wilcoxon signed rank test.

^d^SDQ: Strengths and Difficulties Questionnaire.

#### Differences in Anxiety and Depression Symptoms by Age, Gender, and Ethnicity

Few differences in the mean total outcome scores were found for the GAD-7 and MFQ when examined by age, gender, and ethnic group. Almost all groups showed improved anxiety and depression symptom scores at stage 3. The exceptions were Black British (5/37, 14%) and mixed-heritage (3/37, 8%) ethnic groups, whose GAD-7 mean scores increased slightly between stages 1 and 3 (from 13.0 to 13.3 and 11.3 to 13.0, respectively). Black British participants also had a slightly increased MFQ mean score at stage 3 (from 13.0 to 13.3). The greatest improvements in mean scores were a mean reduction of –5.0 (SD 9.8) in the GAD-7 for White other individuals (3/37, 8%) and mean reductions in the MFQ of –5.3 (SD 5.3) for male individuals (11/37, 30%), –6.0 (SD 11.3) for White other individuals (3/37, 8%), and –4.4 (SD 7.8) for those aged 18 to 25 years (25/37, 68%).

## Discussion

### Principal Findings

This paper details the development and evaluation of the Clear Fear app. The main findings on usability, acceptability, safety, and effectiveness were positive overall. The app was effective in reducing probable symptoms of anxiety and depression and emotional and behavioral difficulties. Therefore, this appears to support the hypothesis that a self-guided mobile cognitive behavioral app can improve anxiety symptoms and be acceptable to young people over a period of 9 weeks. However, the limitations of this study (outlined in a later section) should be noted when interpreting these findings.

A post hoc power analysis was conducted using G*Power (version 3.1.9.7) to check whether the sample size used in this study was large enough to facilitate sufficient statistical power for the analyses conducted [18]. The sample of 48 participants at baseline (with a medium effect size of 0.5 and an α of .05) and the sample at stage 3 (follow-up; n=37) provided 90% power and 80% power, respectively. Therefore, the study was adequately powered for the statistical analyses conducted.

### Comparison With Prior Work

#### Usability

Certain parts of the Clear Fear app were frequently used both at stage 2 and stage 3, including Anxiety Types, Clear Your Fear, and Self-Monitoring, features that perhaps targeted participants’ specific needs and symptoms. Over three-quarters of participants at stage 3 (31/37, 84%) reported the Clear Fear app to be easy to use. Patterns of Clear Fear app use remained broadly similar at stages 2 and 3, demonstrating consistent use throughout the study. Other similar studies have reported high use of mental health apps among young people during the first week, which often declines over time [19,20].

A very small minority of participants (5/48, 10%) found the Clear Fear app difficult to use. The app contains many different features and activities, perhaps too many, which, for a minority of participants, made it confusing to navigate and, subsequently, less useful and helpful when trying to find or retrieve parts. The information given and some of the requirements, such as writing down goals and ways to achieve them, may be overwhelming for some. Striking the right balance between ease of use and informativeness is important [21]. Therefore, based on the feedback from a small minority of study participants, future updates of the Clear Fear app could ensure easier navigation and direct access to features if required.

Popular and frequently used features of the Clear Fear app included Anxiety Types, Clear Your Fear, and Self-Monitoring. This appeared to show some participants’ preference for dealing head-on with their feelings of anxiety and fear. The self-monitoring feature provides important emotional awareness to track and reflect on feelings and emotions to identify which promote positive or negative moods [19]. Previous research has also shown that self-monitoring mood is commonly included in many mobile mental health apps for young people and is the most liked and engaged with section [22,23].

#### Qualitative Feedback

The key themes derived from the open-ended responses to the Clear Fear app user questionnaire revealed which aspects participants found useful and what they liked and disliked. The Clear Fear app’s approach to tackling feelings of anxiety and fear appeared beneficial. The exercises (breathing, meditation, and journal writing) within the Clear Fear app were also beneficial in helping some participants calm down from a panic attack. These exercises and other distraction strategies are important for managing anxiety. Some participants also suggested adding videos to watch, calming music, games, and someone speaking reassuring words, which could potentially enhance the Clear Fear app. Video games have been shown to be a useful therapeutic distraction, which helps address and treat symptoms of anxiety [24].

When selecting mobile mental health apps, young people have been found to prefer those that are accessible, secure, and evidence based [25]. The Clear Fear app meets these important criteria.

#### Safety

The Clear Fear app also appeared safe to use, with nearly three-quarters of participants (31/37, 84%) reporting that it offered a lot or some reassurance when feeling anxious or fearful. These reports were confirmed by the pre– and post–follow-up scores for the outcome measures. The “immediate help” feature was an important reminder for some participants to seek help when they needed it while in the meantime providing information and practical strategies for dealing with their anxiety and fear. Encouragingly, over half (21/37, 57%) of the participants reported that the Clear Fear app helped them speak to someone about their anxiety.

#### Effectiveness

At the beginning of this study, just under half (23/48, 48%) of the participants met the threshold for probable symptoms of anxiety disorder, over half (27/48, 56%) had probable depression, and three-quarters (36/48, 75%) obtained a score of *very high* for emotional and behavioral difficulties. By the end of the study, this had decreased to just under a quarter (8/37, 22%) of participants meeting the threshold for probable anxiety disorder, the same proportion (8/37, 22%) for probable depression, and just under two-thirds (23/37, 62%) scoring *very high* for emotional and behavioral difficulties. Consequently, the Clear Fear app appeared to significantly reduce scores for probable anxiety disorder (*P*=.01), probable depression (*P*=.02), and behavioral and emotional difficulties (*P*<.001) after 8 weeks of use. Moreover, at stage 3 of this study, far fewer participants met the threshold for symptoms of probable anxiety disorder and depression in particular.

These results add to the limited available evidence of the effectiveness of app interventions targeting anxiety and depression in young people and confirm previous findings that such apps, according to one review, can produce small to medium positive effect sizes [26]. Despite being distinct conditions, there is a strong relationship between anxiety and depression, which can coexist in young people [27]. This may explain the improvement in depression scores found in this study even though the Clear Fear app is designed for the management of anxiety symptoms. This improvement may be due to several reasons. Anxiety and depression often share common risk and maintaining factors, and addressing anxiety may help with some of the factors that are common to depression. Lowered anxiety may also help improve a person’s self-esteem and confidence; enhance social connection; and help with focus, purpose, and application to work, all contributing to enhanced mood.

#### Differences by Age, Gender, and Ethnic Group

Generally, the Clear Fear app revealed few differences across the age, gender, and ethnic groups examined. However, the differences found highlighted the need to improve the app’s navigation and refine less popular features for Black British and younger users. Interestingly, Black British users had slightly increased scores at follow-up for anxiety and depression symptoms, which underlines the need for more culturally appropriate content.

### Limitations

The small sample of participants prevented further statistical tests to compare differences among age, gender, and ethnic groups. However, such differences were further examined in terms of mean outcomes and qualitative feedback (a strength of this study) to understand the user experiences of the Clear Fear app according to age, gender, and ethnicity.

The Clear Fear app is suitable for ages of ≥11 years; however, participants in the study were aged between 16 and 25 years, making the study findings generalizable only to this age group. Children and adolescents may respond differently to the Clear Fear app layouts, interaction styles, and content than those aged 16 to 25 years.

It is difficult to know what biases may have been introduced during recruitment, for example, how participants self-selected to take part in the study, the different avenues of information (eg, how participants found out about the study and the Clear Fear app), and their motivation to take part. The offer of an incentive (a shopping voucher) could have led to some form of selection bias regarding those who took part in the study. However, the attrition rates were low, with over three-quarters of participants (37/48, 77%) remaining in the study at follow-up, preventing selective biases associated with high dropout rates.

The follow-up of 8 weeks was a relatively short period, and a lack of a control group also presents further limitations in the study design. However, this exploratory uncontrolled pretest-posttest design provides promising initial findings; some understanding of how the Clear Fear app was used; its acceptability; and potential effects on reducing symptoms of anxiety, depression, and emotional and behavioral difficulties. This study was also adequately powered to conduct significance tests on the main outcome after carrying out a post hoc G*Power calculation.

### Future Research

Future studies could benefit from a more robust evaluation design with a larger and more diverse sample. A randomized controlled trial with one or more comparison groups (including a control group) would help determine causality and control for potential confounders and other external influencing factors. This combined with recruiting participants from a wider representation, including children and adolescents (between the ages of 11 and 15 years), could extend the generalizability of this study’s findings. Incorporating clinician-administered outcome measures and extending the follow-up period beyond 8 weeks would enhance understanding of the Clear Fear app’s effectiveness and user engagement over time (eg, over 3, 6, and ≥12 months). Future studies could also help determine how and why the Clear Fear app helped with symptoms of depression.

Future studies could also examine the Clear Fear app’s effectiveness across different cultural contexts to reveal how cultural norms and societal pressures impact user engagement and outcomes. This could help identify any necessary adaptations to the app’s content or functionality to make it culturally sensitive and relevant.

Similarly, future studies could explore how the Clear Fear app can be integrated with specialist mental health services. This could involve studying the app’s role in supporting young adults on treatment waiting lists through enhancing engagement, complementing face-to-face therapy, being administered as a stand-alone intervention, and helping reduce increased workloads for health care providers. Given the need for ongoing support following discharge and in preventing relapse, the Clear Fear app may also have a function in helping young adults stay well.

In addition, further research could focus on understanding the perceptions and acceptance of digital health tools among both patients generally and clinicians in various health care settings, which may vary widely based on local practices and resources. This research will help refine the development of digital interventions, ensuring that they are both accessible and effective for diverse populations and integrated within existing health care systems.

### Conclusions

The findings indicate that the Clear Fear app is an accessible, easy-to-use, safe, and effective early intervention for young people experiencing mild to moderate anxiety and depression. Therefore, this study appears to support the hypothesis that a self-guided mobile cognitive behavioral app can improve anxiety symptoms and be acceptable to young people over a period of 9 weeks. However, it is important to note this study’s limitations, which may restrict the validity and generalizability of these findings, particularly until further research is conducted with larger samples of young people and specifically with children aged between 11 and 15 years to continue to assess the Clear Fear app’s functioning and use. It is also important to examine its effectiveness over a longer period, using more robust and possibly alternative methodologies to better understand its use and compliance. The quantitative and qualitative feedback from participants also provide important information for further improvements to the Clear Fear app through future updates.

## Abbreviations

CBT: cognitive behavioral therapy
GAD-7: 7-item Generalized Anxiety Disorder scale
MFQ: Mood and Feelings Questionnaire
SDQ: Strengths and Difficulties Questionnaire

## Data Availability

The datasets generated and analyzed during this study are not publicly available due to conditions outlined as part of the participants’ informed consent, as well as the data compliance regulations of stem4; however, they may be available from the corresponding author on reasonable request.
